# Favorable Outcomes of Revision Hip Arthroscopy Irrespective of Whether Index Surgery was Performed by the Same Surgeon or a Different Surgeon

**DOI:** 10.5435/JAAOSGlobal-D-21-00107

**Published:** 2021-12-09

**Authors:** Hari K. Ankem, Samantha C. Diulus, Cynthia Kyin, Andrew E. Jimenez, David R. Maldonado, Payam W. Sabetian, Benjamin R. Saks, Ajay C. Lall, Benjamin G. Domb

**Affiliations:** From the American Hip Institute Research Foundation, Des Plaines (Dr. Ankem, Diulus, Kyin, Dr. Jimenez, Dr. Maldonado, Dr. Sabetian, Dr. Saks, Dr. Lall, Dr. Domb); AMITA Health St. Alexius Medical Center, Hoffman Estates (Dr. Saks, Dr. Lall, Dr. Domb); and the American Hip Institute, Des Plaines (Dr. Lall, Dr. Domb), IL.

## Abstract

The purpose of this study was to compare minimum 2-year patient-reported outcomes (PROMs) after revision hip arthroscopy between two different patient cohorts who had undergone primary hip arthroscopy with the same surgeon (SS) and a different surgeon (DS). We hypothesized no difference in clinical outcomes between the groups despite differences in intraoperative findings based on the surgical decision making in a revision setting at a high-volume center.

**Methods:**

Between January 2012 and August 2017, 71 SS patients were matched for age, sex, body mass index, and follow-up to 71 DS patients. Modified Harris hip score (mHHS), nonarthritic hip score, and hip outcome score—sports-specific subscale (HOS-SSS) were collected prospectively. The minimal clinically important difference was calculated for mHHS and HOS-SSS.

**Results:**

All the DS patients had labral tears, and 94.4% had femoroacetabular impingement from residual bony deformity (*P* < 0.001). The SS and DS groups demonstrated significant and comparable improvement in mHHS (Δ = 18.3 ± 21.5 versus 19 ± 20.1; *P* = 0.837), nonarthritic hip score (Δ = 18.8 ± 18.8 versus 18.2 ± 18.8; *P* = 0.850), and HOS-SSS (Δ = 22 ± 27.4 versus 17.5 ± 28.1; *P* = 0.275). The rates of achieving minimal clinically important difference for mHHS and HOS-SSS were similar. Furthermore, the need for revision surgery and conversion to total hip arthroplasty were comparable (*P* = 0.228 and *P* = 0.383).

**Conclusions:**

Patients undergoing revision hip arthroscopy reported notable and comparable improvement in multiple patient-reported outcomes at a minimum 2-year follow-up, irrespective of intraoperative findings or primary source of patient pool.

Arthroscopic treatment for femoroacetabular impingement (FAI) syndrome with labral débridement or repair has resulted in notable long-term improvements in patient-reported outcomes (PROs) and satisfaction.^1,2^ Ganz et al^3^ proposed that early surgical intervention for the treatment of FAI may not only relieve symptoms but also decelerate the progression of degenerative processes in young patients. In addition, the effect of arthroscopic intervention on PROs and degenerative changes has been shown to be longitudinal, with excellent long-term survivorship in patients with labral tears and low-grade cartilage damage.^4^ However, despite these promising outcomes, failures do occur. Residual or unaddressed structural deformities of the hip, such as cam and pincer morphologies, adhesive capsulitis, unaddressed hip microinstability, and underlying osteoarthritis, are commonly associated with failure after hip arthroscopy.^5,6,7,8^

Hip arthroscopy as a technique in itself has a steep learning curve and with an exponential rise in the number of newly trained surgeons performing this procedure; paved the way for increased primary and revision hip arthroscopies.^9,10^ Novel revision capsulolabral treatment options enabled subsequent arthroscopy in select patients to address pathology that was overlooked or incompletely addressed during the primary surgery.^11,12,13,14^ Given the high volume of arthroscopic hip-preserving procedures done annually in the United States, a percentage of patients remain symptomatic with suboptimal PROs.^15,16^ In addition, a notable proportion of active patients attempt to return to sport or activity at their preinjury level, potentially increasing the risk of reinjury.^17^ When pain persists or recurs, patients must decide whether they will return to the surgeon who conducted their index surgery or seek a second opinion. Differences in index surgeon come along with differences in surgical technique, and therefore, the decision to labral débridement versus repair versus reconstruction made during the primary procedure. With this in mind, we ventured to explore the outcomes after revision hip arthroscopy in a high-volume setting^10,11,18,19^ to see whether it is possible for patients inherited from outside surgeons to achieve uniform and comparable outcomes with those who chose to undergo their secondary surgery with the same surgeon (SS).

The purpose of this study was to compare minimum 2-year PROs after revision hip arthroscopy in a group who underwent primary hip arthroscopy with labral treatment at the same institution (same surgeon [SS] group) with a cohort who had their primary arthroscopy at an outside institution (different surgeon [DS] group). We hypothesized no difference in clinical outcomes between both groups despite differing intraoperative findings.

## Methods

### Participation in the XXX Hip Arthroscopy Registry

Despite the novel investigation presented in this study, data on some patients in this study have been included in other studies.^20^ All data collection received institutional review board approval.

### Patient Inclusion and Data Collection

**Figure 1:**
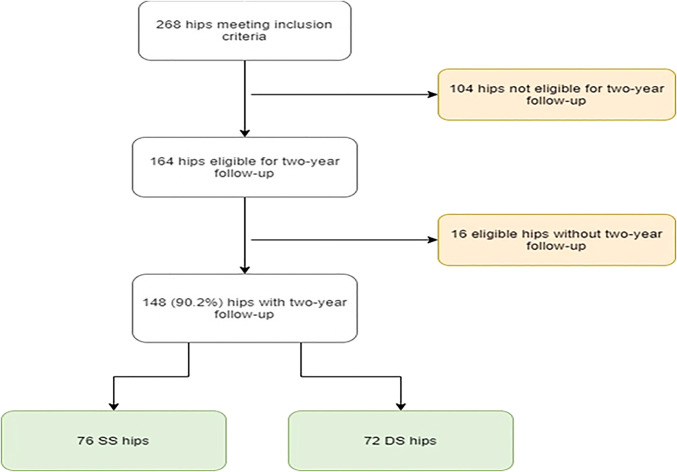
Flowchart showing patient selection.

Data were prospectively collected and retrospectively reviewed for all patients undergoing revision hip arthroscopy by the senior surgeon (XXX) from January 2012 to August 2017, after having their index procedure with the senior surgeon (same surgeon group) or with an outside surgeon (DS group). This study included all patients with minimum 2-year outcomes, including Modified Harris hip score (mHHS) (as the primary outcome measure), along with nonarthritic hip score (NAHS), and hip outcome Score—sports-specific subscale (HOS-SSS), and a visual analog scale (VAS) for pain and satisfaction. Patients were excluded if they had a previous hip condition (such as Ehlers-Danlos syndrome, slipped capital femoral epiphysis, Legg-Calve-Perthes disease, or pigmented villonodular synovitis), incomplete radiographic data, or Tönnis grade >1. Primary and secondary end points were defined as revision hip arthroscopy and total hip arthroplasty (THA), respectively. Patient selection is shown in Figure 1.

### Matching Process

Matching was done on the logit of the propensity score using a nearest-neighbor (Euclidean distance) match algorithm. SS patients were matched to DS patients in a 1:1 ratio using greedy matching without replacement. Consequently, if an SS patient was matched to a DS patient, they were not available to be matched to another DS patient. In the literature, this method has been established as the ideal method for estimating differences between treatment groups.^21^ The SS and DS groups were matched on the basis of age at surgery, sex, body mass index, and follow-up time.

### Imaging

Three plain radiographic views were used to collect pertinent imaging data, including the anterosuperior pelvic view, the 45° Dunn view,^22^ and false profile view,^23^ to determine lateral center edge angle,^24^ lateral joint space, anterior center edge angle, and alpha angle.^25^ The presence of cam morphology was indicated by an alpha angle greater than 55°.^25,26^ The senior surgeon (XXX) made all measurements using a picture archiving and communication system. To evaluate for a labral tear and articular cartilage damage, magnetic resonance imaging was done on each patient.

### Surgical Technique

All revision hip arthroscopic procedures were done with the patient in the supine position through a minimum of two portals (anterolateral and mid-anterior). The indications for revision arthroscopy were predominantly labral tears with FAI (from residual cam, pincer, or combined morphology) causing mechanical symptoms and failure of conservative treatment. At the beginning of the procedure, a diagnostic arthroscopy was undertaken. First, the state of the ligamentum teres was noted using the classification scales of Domb et al, as well as Gray and Villar.^27,28^ The labrum was then assessed for the presence and degree of tearing using the classification system of Seldes et al.^29^ The cartilage of the acetabulum and femoral head was subsequently surveyed for defects and lesions using the Outerbridge^30^ and acetabular labrum articular disruption grading systems.^31^ The location and size of labral tears and chondral damage were evaluated using a 5-mm probe and recorded in mm^2^ using the clockface method.

Residual bony deformities were corrected using fluoroscopic guidance. An acetabuloplasty and femoroplasty were used to correct pincer and cam morphologies, respectively. Labral repair was done when possible; however, some cases required selective débridement to achieve a stable labrum while preserving as much of the labrum as possible. In the presence of an irreparable tear, labral reconstruction was done using either autograft or allograft gracilis hamstring tendon.^32,33^ If full-thickness cartilage defects were discovered, the area was first débrided to create stable borders before performing microfracture according to the technique described by Maldonado et al.^34^

### Postoperative Rehabilitation Protocol

Postoperative rehabilitation was adjusted according to intraoperative procedures. However, all protocols consisted of an X-Act range-of-motion brace (DJO Global) and flatfoot weight bearing (20 lbs.) on crutches for 2 or 6 weeks.^35^ Patients undergoing labral reconstruction or microfracture were prescribed the longer duration. Regardless of intraoperative procedures done, physical therapy began on the first day after surgery.

### Surgical Outcomes

All patients undergoing hip arthroscopy were assessed preoperatively and postoperatively at 3 months, 1 year, 2 years, and annually thereafter using the mHHS, NAHS, HOS-SSS, and VAS. Four questionnaires were used because of the lack of conclusive evidence for the use of a single PRO measure for patients undergoing hip arthroscopy.^36^ Pain was quantified on a VAS from 0 to 10, with 10 being the worst. Similarly, a 0 to 10 scale was used to assess patient satisfaction, with 10 being the best in this scenario. Additional postoperative surveys included the international hip outcome tool-12, short form 12 physical and mental, and veterans RAND 12-item health survey physical and mental. The institution's database automatically computed, stored, and encrypted these values. Using the method described by Norman et al.,^37^ minimal clinically important difference (MCID)^38^ for mHHS and HOS-SSS was calculated for both groups. Survivorship rates were determined using the rates of revision arthroscopy and conversion to THA that were recorded during the collection of the follow-up data.

### Statistical Analysis

An a priori power analysis was done before statistical analysis to determine the minimum number of hips required to achieve 80% power. Under the assumption that a mean eight-point difference in mHHS would be statistically significant when comparing groups, it was determined that each group required a minimum of 51 patients to demonstrate differences between groups. To calculate significance between preoperative and postoperative groups, a paired Student *t*-test was used. When comparing measurements between the same surgeon revision group and the group who transferred from an outside practice, a Mann-Whitney test for independent samples was used. When comparing categorical data, a chi-square test was used. *P* values <0.05 were considered statistically significant. The change from preoperative to postoperative scores (delta) was calculated and compared between the groups. Aside from propensity score matching, all statistical analyses were done with Microsoft Excel (Redmond) and the Real-Statistics add-in.

## Results

### Patient Demographics

Seventy-six patients had their primary hip arthroscopy with the senior surgeon (same surgeon), and 72 patients had their primary hip arthroscopy with a DS at an outside practice (Figure 1). After propensity score matching using the aforementioned criteria, 71 SS hips (70 patients) and 71 DS hips (70 patients) comprised each group. No significant differences were observed in age at surgery, sex, body mass index, laterality, follow-up time, or time to revision. These findings are outlined in Table 1.

**Table 1:** Demographic Data for the SS and DS Groups

	SS	DS	*P*
Age, years	35 ± 12.5	31.2 ± 10.4	0.099
BMI, kg/m^2^	26 ± 4.2	26.6 ± 4.8	0.432
Sex			0.201
Male	18 (25.3)	25 (35.2)	
Female	53 (74.6)	46 (64.8)	
Laterality			0.313
Right	35 (49.3)	41 (57.7)	
Left	36 (50.7)	30 (42.3)	
Follow-up time, months	41.5 ± 18.5	46.9 ± 21	0.140
Time to revision, months	22.5 ± 17.9	28.3 ± 33.8	0.226

BMI = body mass index, DS = different surgeon, SS = same surgeon

Values reported as n (%) or mean ± SD; n = sample size.

### Radiographic Findings

Radiographs reflected that there was a high proportion of hips graded Tönnis 0 in both groups, with 59 hips (83.1%) in the SS group and 59 hips (83.1%) in the DS group (*P* > 0.999). The presence or absence of cam morphology was indicated by the alpha angle, with the mean measurement being 47.6° in the SS group and 56.2° in the DS group (*P* < 0.001), indicating a higher prevalence of cam morphology in the latter cohort. Table 2 outlines these findings.

**Table 2:** Radiographic Findings in the SS and DS Groups

	SS	DS	*P*
Tönnis grade			>0.999
0	59 (83.1)	59 (83.1)	
1	12 (16.9)	12 (16.9)	
LCEA (deg)	29.2 ± 5.3	29.8 ± 5.3	0.292
Acetabular inclination (deg)	5.4 ± 4.1	5.8 ± 4.4	0.617
Lateral joint space (cm)	0.40 ± 0.1	0.41 ± 0.1	0.368
ACEA (deg)	30.5 ± 7.8	31.2 ± 5.4	0.809
Alpha angle (deg)	47.6 ± 11.8	56.2 ± 12.5	**<0.001**

ACEA = anterior center edge angle, DS = different surgeon, LCEA = lateral center edge angle, SS = same surgeon

Bold represents statistical significance; values reported as n (%) or mean ± SD; n = sample size.

### Intraoperative Findings and Surgical Procedures

Labral tears were extremely prevalent in the DS group at 100% compared with 72.4% in the SS group (*P* < 0.001). The combined type 1 and 2 labral tears were found in 14.0% of the SS group and 43.7% of the DS group, respectively (*P* < 0.001). FAI was also more prevalent in the DS group, with 94.4% patients having significant cam and/or pincer morphology (*P* < 0.0001). Moreover, in the DS group, 93.0% and 60.6% had cam and pincer deformities, respectively (*P* < 0.0001 and *P* = 0.0005). Furthermore, acetabular cartilage damage, classified using both acetabular labrum articular disruption and acetabular Outerbridge, was more common in the DS cohort (*P* = 0.044 and *P* = 0.006, respectively). Intraoperative findings are provided in Tables 3 and 4.

**Table 3:** Intraoperative Findings in the SS and DS Groups

	SS	DS	*P*
Seldes			**<0.001**
0	21 (29.6)	0 (0)	
1	8 (11.3)	13 (18.3)	
2	32 (45.1)	27 (38.0)	
1 and 2	10 (14.0)	31 (43.7)	
ALAD			**0.044**
0	23 (32.4)	10 (14.0)	
1	18 (25.4)	26 (36.7)	
2	20 (28.2)	16 (22.5)	
3	7 (9.9)	13 (18.3)	
4	3 (4.1)	6 (8.5)	
Acetabular Outerbridge			**0.006**
0	23 (32.4)	9 (12.7)	
1	18 (25.4)	25 (35.2)	
2	21 (29.6)	15 (21.1)	
3	6 (8.5)	11 (15.5)	
4	3 (4.1)	11 (15.5)	
Femoral head Outerbridge			0.420
0	63 (88.7)	56 (78.9)	
1	0 (0)	2 (2.8)	
2	2 (2.8)	4 (5.7)	
3	4 (5.7)	7 (9.8)	
4	2 (2.8)	2 (2.8)	
LT percentile class: Domb			0.318
0: No tear	48 (67.5)	40 (56.3)	
1: 0% to 50%	13 (18.3)	19 (26.9)	
2: 50% to <100%	6 (8.5)	10 (14.0)	
3: 100%	4 (5.7)	2 (2.8)	

ALAD = acetabular labrum articular disruption, DS = different surgeon, FAI = femoroacetabular impingement, LT = ligamentum teres, SS = same surgeon

Bold represents statistical significance; values reported as n (%); n = sample size.

**Table 4:** Presence or Absence of Intraarticular Pathologies

	SS	DS	*P*
Labral tear			**<0.001**
Yes	50 (70.4)	71 (100)	
No	21 (29.6)	0 (0)	
LT tear			0.167
Yes	23 (32.4)	31 (43.7)	
No	48 (67.6)	40 (56.3)	
FAI			
Yes	41 (57.7)	67 (94.4)	**<0.001**
No	30 (42.3)	4 (5.6)	
Cam	39 (54.9)	66 (93.0)	**<0.001**
Pincer	22 (31.0)	43 (60.6)	**<0.001**

DS = different surgeon, LT = ligamentum teres, FAI = femoroacetabular impingement

Bold represents statistical significance; values reported as n (%); n = sample size.

### Surgical Procedures

Tables 5 and 6 summarize the variety of surgical procedures done in both groups. Labral treatment, defined as either repair or reconstruction, was required in 35.2% of the SS group and 71.8% of the DS group (*P* < 0.001). An acetabuloplasty of 2 mm or greater was done on 26 SS patients (36.7%) and 47 DS patients, (66.2%) (*P* < 0.001). Treatment of cam impingement with femoroplasty was required in 57.7% and 94.4% of patients in the SS and DS group, respectively (*P* < 0.001). The proportion of patients requiring capsular plication or repair was also greater in the DS group at 56.3% (compared with 32.4%, *P* = 0.004). In addition, microfracture of the acetabulum was done more frequently in the DS group, with 14.1% of patients requiring this procedure (compared with 1.4% in the SS group, *P* = 0.005).

**Table 5:** Surgical Procedures Done in the SS and DS Groups

	SS	DS	*P*
Labral treatment			**<0.001**
None	15 (21.1)	0 (0)	
Débridement	31 (43.7)	20 (28.2)	
Repair	7 (9.9)	33 (46.4)	
Reconstruction	18 (25.3)	18 (25.4)	
Capsular treatment			**0.004**
Repair/plication	23 (32.4)	40 (56.3)	
Release	48 (67.6)	31 (43.7)	
Microfracture			
Acetabulum	1 (1.4)	10 (14.1)	**0.005**
Femoral head	2 (2.8)	1 (1.2)	0.560
LT treatment			0.438
None	64 (90.1)	61 (85.9)	
Débridement	7 (9.9)	10 (14.1)	

DS = different surgeon, LT = ligamentum teres, SS = same surgeon

Bold represents statistical significance; values reported as n (%); n = sample size.

**Table 6:** Presence or Absence of Labral, Capsular, and FAI Treatment

	SS	DS	*P*
Labral repair/reconstruction			**<0.001**
Yes	25 (35.2)	51 (71.8)	** **
No	46 (64.8)	20 (28.2)	
Capsular plication/repair			**0.004**
Yes	23 (32.4)	40 (56.3)	
No	48 (67.6)	31 (43.7)	
Acetabuloplasty	26 (36.7)	47 (66.2)	**<0.001**
Femoroplasty	41 (57.7)	67 (94.4)	**<0.001**

DS = different surgeon, SS = same surgeon

Bold represents statistical significance; values reported as n (%); n = sample size.

### Patient-reported Outcomes

**Figure 2:**
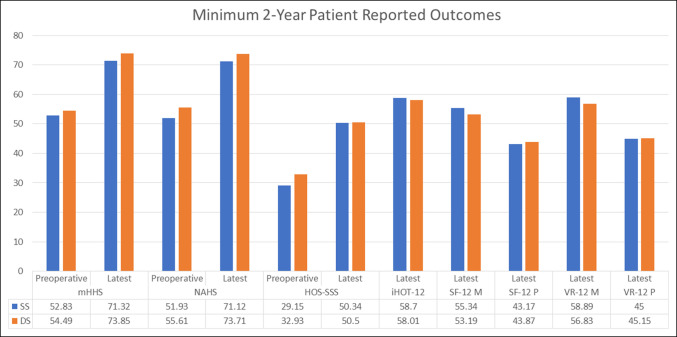
Graph showing preoperative and minimum 2-year patient-reported outcomes. DS = different surgeon, HOS-SSS = hip outcome score—sports-specific subscale, iHOT-12 = international Hip Outcome Tool 12, mHHS = Modified Harris hip score, NAHS = nonarthritic hip score, SF-12 M and P = short form 12 mental and physical, SS = same surgeon, VR-12 M and P = veterans RAND 12 mental and physical

**Figure 3:**
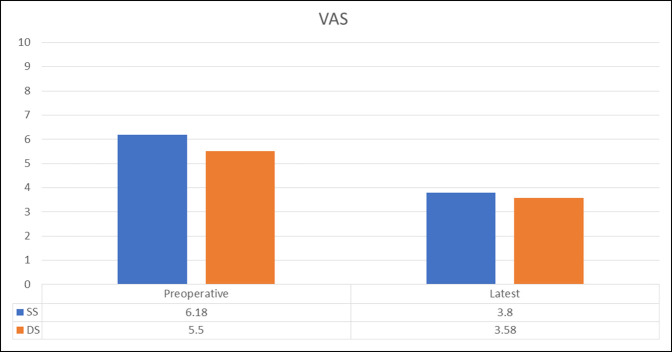
Graph showing preoperative and minimum 2-year VAS scores for the SS and DS groups. DS = different surgeon, SS = same surgeon, VAS = visual analog scale

**Figure 4:**
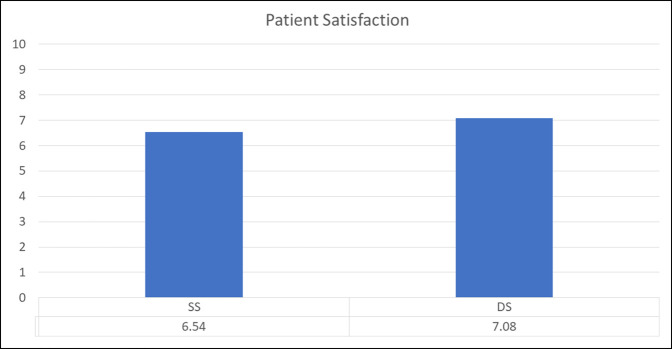
Graph showing SS and DS patient satisfaction scores at the most recent follow-up. DS, different surgeon, SS, same surgeon

Table 7 summarizes PROs measured preoperatively and at a minimum of 2 years postoperatively. All patients in both the SS and DS groups demonstrated statistically significant improvement from preoperative to the latest follow-up in mHHS, NAHS, HOS-SSS, and VAS scores. When comparing mean preoperative and latest scores for all the aforementioned questionnaires, both groups faired similarly (*P* > 0.05) (Figures 2–4).

**Table 7:** Improvement in Patient-reported Outcomes and Patient Satisfaction at the Latest Follow-up for the SS and DS Groups

	SS	DS	*P*
mHHS			
Pre	52.8 ± 13.7 (13 to 96)	54.5 ± 13.9 (24 to 92)	0.477
Latest	71.3 ± 20.8 (23 to 100)	73.9 ± 18.8 (32 to 100)	0.646
*P* value	**< 0.0001**	**< 0.0001**	
Delta	18.3 ± 21.5 (−28-61)	19 ± 20.1 (−52-70.6)	0.837
NAHS			
Pre	51.9 ± 16.4 (5 to 96.3)	55.6 ± 16 (14 to 91.3)	0.178
Latest	71.1 ± 20.2 (17.5 to 100)	73.7 ± 17.5 (27.5 to 100)	0.646
*P* value	**<0.0001**	**<0.0001**	
Delta	18.8 ± 18.8 (−18.8-61.3)	18.2 ± 18.8 (−21.3 to 61.3)	0.850
HOS-SSS			
Pre	29.2 ± 19.2 (0 to 80.6)	32.9 ± 22.4 (0 to 100)	0.424
Latest	50.3 ± 25.4 (0 to 100)	50.5 ± 25.8 (2.8 to 100)	0.972
*P* value	**<0.0001**	**<0.0001**	
Delta	22 ± 27.4 (−50 to 81.9)	17.5 ± 28.1 (−83.3 to 84.4)	0.275
VAS			
Pre	6.2 ± 1.9 (2 to 10)	5.5 ± 2.1 (0 to 10)	**0.046**
Latest	3.8 ± 2.7 (0 to 10)	3.6 ± 2.3 (0 to 8.1)	0.913
*P* value	**<0.0001**	**<0.0001**	
Delta	−2.3 ± 3.2 (−8 to 6.9)	−1.9 ± 2.5 (−9 to 4)	0.359
iHOT-12	58.7 ± 27.4 (4.3 to 100)	58 ± 22.2 (11 to 98.8)	0.806
SF-12			
Mental	55.3 ± 9.2 (26.3 to 69.4)	53.2 ± 10.4 (24.8 to 66.1)	0.334
Physical	43.2 ± 10.2 (21.3 to 56.8)	43.9 ± 9.7 (21.1 to 60.6)	0.842
VR-12			
Mental	58.9 ± 8.9 (30.5 to 69.2)	56.8 ± 11.1 (25.4 to 68.9)	0.454
Physical	45.0 ± 9.8 (20.9 to 57.7)	45.2 ± 9.5 (20.5 to 57.5)	0.991
Satisfaction	6.5 ± 2.9 (0 to 10)	7.1 ± 2.5 (0 to 10)	0.338

DS = different surgeon, HOS-SSS = hip outcome score—sports-specific subscale, iHOT-12= international Hip Outcome Tool-12, mHHS = Modified Harris hip score, NAHS = nonarthritic hip score, SF-12 P and SF-12 M = short form 12 physical and mental, SS = same surgeon, VAS = visual analog scale, VR-12 P and VR-12 M = veterans RAND 12-item health survey physical and mental

Bold represents statistical significance; values reported as mean ± SD.

**Figure 5:**
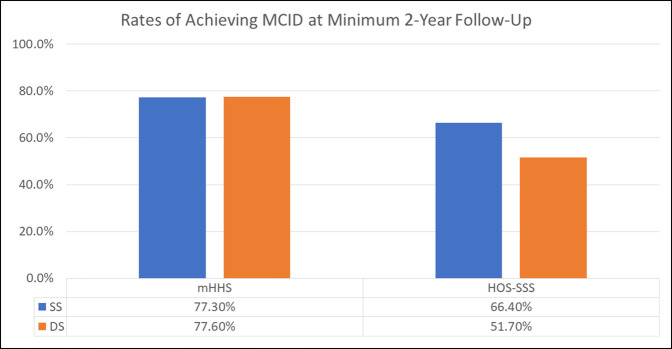
Graph showing the rates of achieving MCID in the SS and DS groups. DS = different surgeon; HOS-SSS = hip outcome score—sports-specific scale; MCID = minimal clinically important difference; mHHS = Modified Harris hip score; SS = same surgeon

MCIDs for mHHS and HOS-SSS were calculated for both groups. Table 8 summarizes these findings. Fifty-one SS patients (77.3%) and 52 DS patients (77.6%) achieved or exceeded MCID for mHHS (*P* = 0.963). Similarly, 39 patients (68.4%) and 30 patients (51.7%) met the MCID threshold value for HOS-SSS in the SS and DS groups, respectively (Figure 5).

**Table 8:** SS and DS Patients Who Achieved MCID for mHHS and HOS-SSS

	SS	DS	*P*
mHHS			
MCID	51 (77.3)	52 (77.6)	0.963
HOS-SSS			
MCID	39 (68.4)	30 (51.7)	0.068

SS, same surgeon; DS, different surgeon; values reported as n (%); n = sample size; Modified Harris hip score (mHHS); hip outcome score—sports-specific subscale (HOS-SSS); minimal clinically important difference (MCID).

### Secondary Procedures

Four patients (5.6%) in the SS group and eight patients (11.3%) in the DS group required re-revision hip arthroscopy (*P* = 0.228) at a mean 27.9 and 19.2 months, respectively (*P* = 0.283). The rate of conversion to total hip replacement was also comparable with eight patients (11.3%) in the SS group and five patients (7.0%) in the DS group (*P* = 0.383). The time to conversion was not statistically significant between groups (*P* = 0.943) (Table 9).

**Table 9:** Rate of Secondary Surgeries in the SS and DS Cohorts

	SS	DS	*P*
Re-revision arthroscopy, n (%)	4 (5.6)	8 (11.3)	0.228
Time to re-revision arthroscopy, mo	27.9 ± 16 (10.4-44.2)	19.2 ± 16.9 (4.3-55.2)	0.283
Total hip replacement, n (%)	8 (11.3)	5 (7.0)	0.383
Time to total hip replacement, mo	26.4 ± 15.3 (2.6-47.5)	31.3 ± 22 (15.1-69.6)	0.943

DS = different surgeon, SS = same surgeon

Values reported as n (%) or mean ± SD (range); n = sample size.

## Discussion

Outcomes of revision hip arthroscopy, with strict indications, seem to be unaffected by markedly different intraoperative findings found within pair-matched cohorts managed at the index by DSs. After index procedures, labral tears were present in 100% of DS patients compared with 70.4% of SS patients. FAI from residual bony deformity was found in 94.4% of DS patients and 57.7% of SS patients, which was significantly different (*P* < 0.001). Both groups demonstrated significant and comparable improvement in mHHS (*P* = 0.850), HOS-SSS (*P* = 0.275), and NAHS (*P* = 0.850) at the minimum 2-year follow-up. Rates of achieving MCID for mHHS and HOS-SSS were similar (*P* = 0.963 and *P* = 0.068, respectively) between the groups. Furthermore, the need for revision surgery and conversion to THA were comparable (*P* = 0.228 and *P* = 0.383). Revision hip arthroscopy procedures seem to do equally well for patient-reported outcomes at the minimum 2-year follow-up, regardless of index procedure indications and revision surgery type.

The literature repeatedly demonstrates that persistent or residual structural deformity in the form of cam-type and/or pincer-type impingement postoperatively is associated with revision surgery.^6,7,39^ In this study, we found a markedly increased amount of undertreated or unaddressed FAI in the DS group, which is consistent with previous studies.^7,8^ In addition, there were notable differences in the DS group, such as correction of FAI, labral tears requiring additional treatment, and capsular plication. Despite these differences, both patient populations were able to achieve similar improvement in outcomes after revision hip arthroscopy. The data demonstrate that the volume-outcome relationship^40^ holds true in the revision hip arthroscopy setting, irrespective of where the index procedure is done.

In an earlier study by Philippon et al,^6^ a 95% incidence of residual FAI was reported in a cohort undergoing revision hip arthroscopy, indicating that persistent impingement was the most common indication for revision hip arthroscopy. A more modest benefit of revision hip arthroscopy, with a lower incidence of missed or incompletely treated FAI (32%), was reported by Aprato et al.^41^ Chondral lesion associated with labral reinjury was the most common finding at revision arthroscopy in a study by Aprato et al.^41^ In contrast to a study by Philippon et al,^6^ only 31% of patients underwent revision for persistent FAI according to this study. These authors commented that this observation may be because of differences in patient population or patient selection. However, the rates of untreated or incompletely addressed FAI in the present investigation, with 93.0% and 60.6% of DS patients presenting with cam impingement and pincer impingement, respectively, reinforce the existing literature findings.

There has been a recent surge in relevant literature reporting on revision hip arthroscopy findings and outcomes which echo our study findings. Larson et al.^8^ compared revision and primary hip arthroscopy cohorts that needed FAI correction and reported good or excellent results in 62.7% of revision cases (mean mHHS improvement of 17.8) compared with 81.7% in primary cases (mean mHHS improvement of 23.4). The findings of our study align with those described here. Both cohorts (SS and DS groups) undergoing revision arthroscopy demonstrated statistically significant improvements in minimum 2-year outcomes. Furthermore, 77.3% and 77.6% of patients in the SS and DS cohorts, respectively, achieved MCID for mHHS, demonstrating clinically significant improvement in addition to statistically significant improvement in PROs. In a recent systematic review that was conducted by Cvetanovich et al,^42^ the authors concluded that the revision hip arthroscopy is most commonly done for residual FAI and is associated with statistically significant and clinically relevant improvements shown in multiple patient-reported clinical outcome scores at the short-term follow-up.

However, it has also been recently shown that the results after revision hip arthroscopy may not be as durable as those after primary hip arthroscopy. Aprato et al.^41^ showed decreasing mHHS and satisfaction at the 3-year follow-up. Similarly, Gwathmey et al questioned the durability of revision arthroscopy outcomes in their study echoing the study findings of Aprato et al.^15,41^ By contrast, our results showed a substantial improvement from preoperative state in the mHHS and HOS-SSS at 2 years, as indicated by the rate of achieving MCID, in most of the revision arthroscopy patients. However, there was no statistically significant difference in the rate of revision hip arthroscopy, time to revision, THA conversion rate, or time to conversion between the two groups in our study. Hence, more research on the durability after revision hip arthroscopy is needed to determine long-term function and success in avoiding THA.

### Strengths

The present investigation is enhanced by a variety of strengths. Our study design is unique in that we could compare two patient cohorts with similar demographics who underwent revision hip arthroscopy by the SS but differed in the index surgeon. To the authors' knowledge, this is the first study to compare patients in this manner. Furthermore, these cohorts were compared after propensity score matching, a process that limited the effect of potentially confounding variables. In addition, an a priori power analysis increased the generalizability of the results. The ceiling effect of a single PRO was avoided by including a variety of PROs that were designed for active patients without arthritic hips. Finally, statistical significance was translated into clinical relevance through the calculation of MCID achievement rates.

### Limitations

Despite the aforementioned strengths, this study is not without limitations. Although a matched-pair design was implemented, the effect of confounding variables could not be completely eliminated because this was a nonrandomized study. In addition, the study design is retrospective, which may have introduced bias. However, all data were collected prospectively, limiting the effect of the retrospective design. Another key limitation is that we are unable to identify the index surgery indications and procedures in the DS group and the interval between surgeries. Additional investigation as to why the outcomes and THA conversion rates were similar when pathologies and treatments were markedly different between DS and SS groups is needed. Another major limitation is that we lack information on the index surgeon in the DS group. Hence, a sampling bias is likely when the volume-outcome relationship is discussed while only examining the work of a high-volume surgeon. Moreover, re-revision rate in the DS group is twice that in the SS group but was not found to be significant. This is possibly due to a type II error.

Furthermore, it is important to note that the field of hip arthroscopy has evolved markedly over the past decade,^10,11,18,19^ causing a shift in preferred capsular and labral treatments.^11,12,43^ Consequently, patients who were treated with labral débridement and capsulotomy without repair earlier in the study period would currently be managed with labral reconstruction and capsular repair/plication.^13,14,44^ The objective clinical findings (both preoperative and intraoperative) and subjective patient-related outcomes lacked correlation. Furthermore, other than stringent indications, it is difficult to extrapolate the reason for comparable outcome findings between these groups based on these subtle differences. Although the minimal change in the outcome scores, that is notable, helps us understand there is subjective symptomatic improvement, this study reiterates the fact that there may be several less understood patient factors that are at play in dictating patient outcomes. It is also important to consider the ceiling effect when looking at outcome scores because the hip joint is known to show improvement even after revision surgeries. There are unknown or less well-understood patient factors related to successful revision hip arthroscopy surgery noted in this study. Because the present investigation examined minimum 2-year outcomes, long-term follow-up studies were needed to determine the durability of the conclusions presented here. Finally, it is possible that the good and comparable outcomes achieved in different patient cohorts may be a product of this single surgeon's wealth of experience; hence, these results may not be generalizable.

## Conclusion

Patients undergoing revision hip arthroscopy reported notable improvement in multiple PROs at the minimum 2-year follow-up. Outcomes of revision hip arthroscopy, with strict indications, seem to be unaffected by markedly different intraoperative findings found within pair-matched cohorts managed at the index by DSs. In addition, the rates of achieving MCID for mHHS and HOS-SSS were similar between groups.
